# Supplementary figures and images for: OSS-DBS: Open-source simulation platform for deep brain stimulation with a comprehensive automated modeling
Source: PLoS Comput Biol. 2020 Jul 6;16(7):e1008023. doi: 10.1371/journal.pcbi.1008023 (PMC7384674; doi:10.1371/journal.pcbi.1008023)

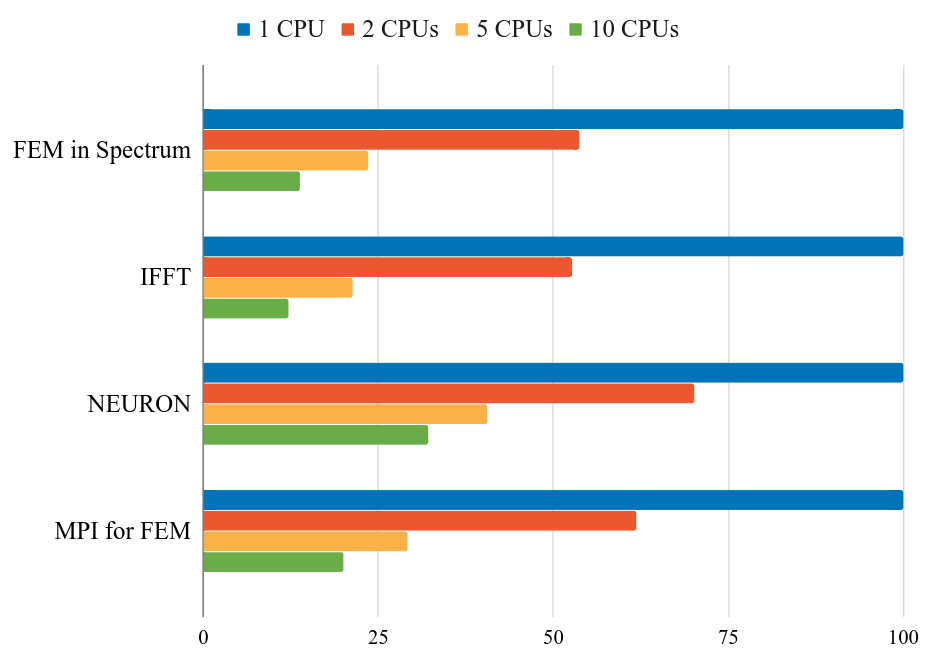

Supplement: S1 Fig — The bars show computational time as % to the single CPU run. (TIF) [file pcbi.1008023.s001.tif]

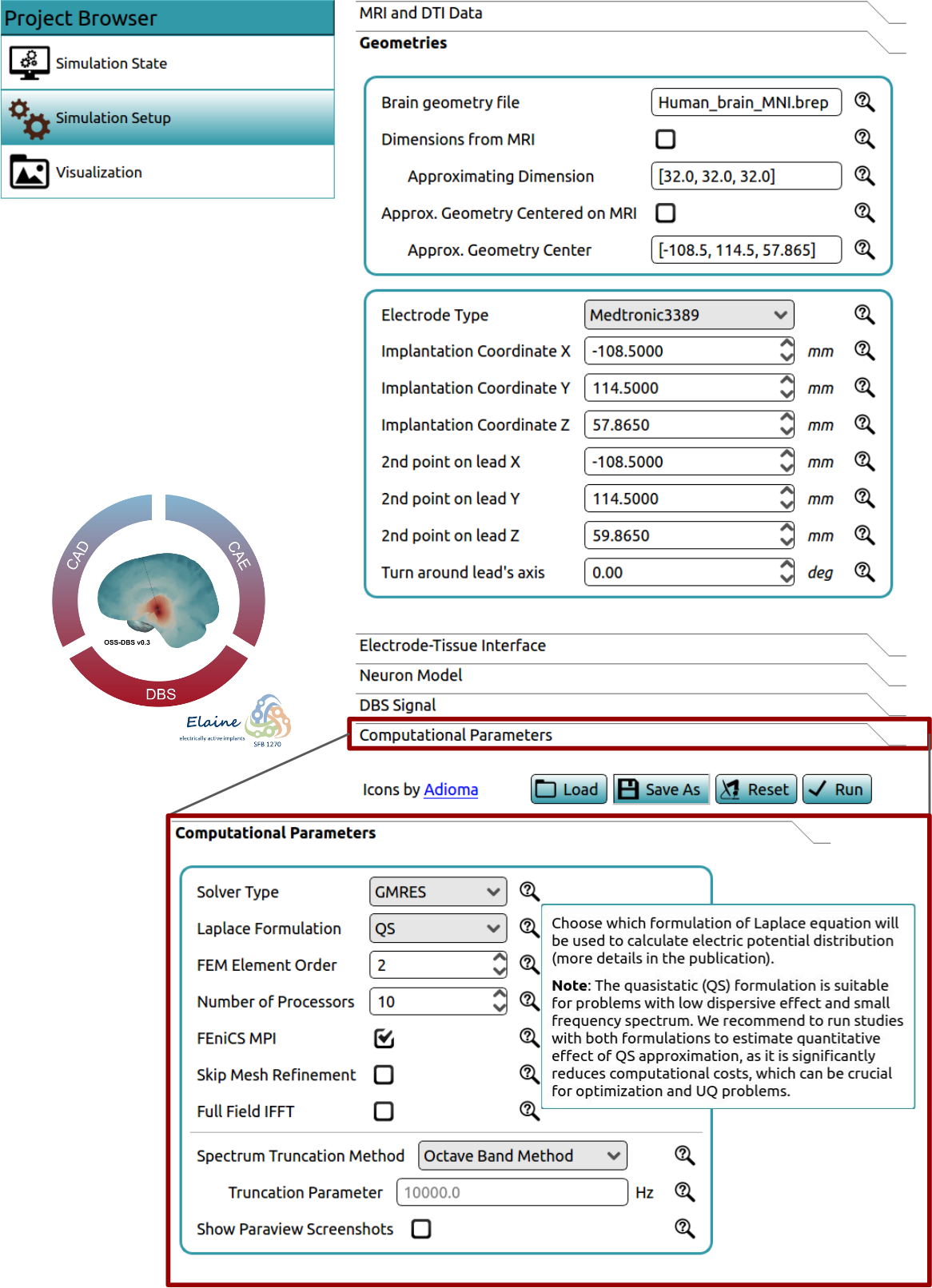

Supplement: S2 Fig — (TIF) [file pcbi.1008023.s002.tif]
