# Supplementary material for: OSS-DBS: Open-source simulation platform for deep brain stimulation with a comprehensive automated modeling
Source: PLoS Comput Biol. 2020 Jul 6;16(7):e1008023. doi: 10.1371/journal.pcbi.1008023 (PMC7384674; doi:10.1371/journal.pcbi.1008023)
Supplement: Attachment — Submitted filename: Butenko_Answers_and_corrections.pdf [file pcbi.1008023.s004.pdf]

Elaine
electrically active implants
SFB 1270

Albert Einstein, 2
18059 Rostock

Date
27.05.2020

Responses to the review comments and other corrections for the software article "OSS-DBS: Open-Source Simulation Platform for Deep Brain Stimulation with a comprehensive automated modeling".

To Reviewer #1:

1) Namely, one open source alternative that is broadly used in the more clinically oriented field (Lead-DBS, www.lead-dbs.org, also cited by the authors) comprises of a much more limited FEM-based VTA model. Thus, a direct exemplary comparison between results from Lead-DBS (say in a single patient) and OSS-DBS could be interesting to the medical community. Are results similar? If they are different, what reasons may apply? Which settings in the larger parameter space of OSS-DBS would lead to similar solutions as in Lead-DBS?

Lead-DBS solves the static formulation of Laplace equation, i.e. with fixed conductivity and without capacitive effect of the tissue. The output of Lead-DBS is volume of tissue activated (VTA) assessed either using a threshold for the derivatives of the electric potential or impedance estimations in case of a monopolar stimulation. While this volume metric might not change drastically when applying a more advanced physics as implemented in OSS-DBS, this representation of VTA poorly describes the DBS effect on the neural tissue, in particular, the action potential generation in axons. And in this case, we indeed observe the influence of the model complexity on the activation rate, especially due to the dispersivity of the brain tissue. The reason why the effect of the model complexity on the activation was not demonstrated in the paper is an employment of the externally developed neuron models (mammalian axon model by McIntyre).

In fact, we established a collaboration with Lead-DBS to combine their medical imaging methods with our field and neuron modelling approaches. So the comparative study you proposed will be available in the combined tool.

2) Are the insulating of the electrodes explicitly modeled? I do think those would have an effect on the shape of the stimulation volume.

The surface of the electrode (except the exposed contacts) is assumed to be a perfect insulator, and we apply the homogeneous Neumann boundary condition to ensure absence of the electric field ($\nabla\phi \cdot n=0$). Modelling the insulator as a body with extremely low conductivity does not yield a

significantly different electric potential distribution, but extremely complicates solution of the FEM problem due to highly heterogeneous material distribution and thus ill-conditioned stiffness matrix. On the other hand, floating conductors indeed disturb the distribution, and, at the moment, they are modelled explicitly.
A comment regarding the homogeneous boundary condition for insulators was added to the manuscript.

3) I think dystonias (plural) in the introduction is a bit uncommon (singular dystonia would be more appropriate here)

Agreed, corrected in the manuscript.

4) St. Jude Medical was bought by Abbott, so maybe specifying electrode types with the novel name is more appropriate.

On Abbott's websites St. Jude naming is used for the electrodes. Internet search for Abbott electrodes also returns St. Jude. So for now, we will use this name to avoid confusion. If the company representative requests a renaming, this will not be a problem.

To Reviewer #2:

General comment from the authors: could you elaborate on the specific problem about access to the repository? Was the webpage unavailable or were the provided credentials not valid? Meanwhile we have uploaded the project to a freely available Github repository (https://github.com/SFB-ELAINE/OSS-DBS), but still it would be good to know for future publications what was the issue.

1) However, the lack of presenting any example regarding the user-interface is one major downside of this manuscript. So this reviewer would like to recommend to the authors to provide usage examples (with some screenshot of the user interface) on supplementary so that readers can get a better idea of how the platform can benefit their research. This aspect was not able to evaluate in terms of easy to use or rapid setup capability which authors claimed, because all link that authors provide was not accessible for the reviewers.

The OSS-DBS tutorial (available in the repository) elaborates on the user-interface interaction. Additionally, we added a screenshot of the GUI to S3 Appendix.

2) In the abstract and introduction, the authors mentioned that this platform 'is suitable for a clinical study.' Still, since this software could be classified as 'clinical decision support' software for deep brain stimulation surgery, authors may need to check about the regulation on this category of the software if there is no issue to describe as 'suitable for clinical use.'

After reviewing legal documents on the issue, we have edited the phrasing in the abstract and *Discussion* section. In *Introduction*, there is no direct reference to the clinical use of OSS-DBS (only *in silico* models as such).

3) While the authors state that this platform provides a highly automated solution, but since there is no real demonstration of platform operation, it is not clear the level of automation that this platform is providing. For example, I am wondering interacting with other GUI software such as Paraview also fully automated and how much time could take on a regular operation task (for example, for the benchmark example) if the user has all parameters in hands.

To get acquainted and test the platform, users are offered a collection of predefined setup dictionaries (additionally to the benchmark models) with the corresponding meta data files. All steps to set up a simulation with the GUI are thoroughly described in the tutorial. After the input dictionary is completed, the platform will conduct all modelling steps autonomously and return the requested output (usually, activation rate in axons or size of VTA assessed from the electric potential derivatives). Furthermore, the platform will generate a number of .pvd and .csv files that can be used for visualization in Paraview. We provide a collection of scripts that process these files and make screenshots available for a display in the GUI. If the platform's software is installed directly in the system, this is also done automatically. However, with the Docker approach, this feature is yet not supported; we are working on the issue. Meanwhile, users can either run the scripts from Paraview or through the terminal, but in both cases Paraview should be installed on the user's system.

However, we would not recommend the benchmark models to test the platform as they are intended to demonstrate the adaptive refinement algorithm, which in these cases takes some iterations and time (more than 90 minutes on a powerful machine).

4) The authors state that the platform offers to the user to choose an electrode geometry from the predefined collection. Does this customizable? If so, could the experimentally measured impedance spectrometer result also be used as input for estimating electrode-tissue interface?

Users can modify the provided electrodes or create their own using a guidance from the repository. However, it might not be that intuitive, especially for users without CAD experience. In this case, we would be happy to add the geometry upon request.

A comment regarding custom electrodes was added to the manuscript.

At the moment, the electrode-tissue interface is modelled simply with an encapsulation layer and a constant-phase element. The reasons why we did not implement more advanced physics are

- Multitude of uncertain parameters to describe the interface
- Application of the current-controlled stimulation that allows to neglect (to a certain extent) the interface effects.

What we could implement in the next version is a direct input of the impedance from the user and a corresponding voltage drop over the interface. The limitation is that the drop will be uniform across the surface. However, the estimation of the electrode-tissue interface parameters lies beyond the scope of the project. In fact, our group also works on the automated extraction of the interface parameters from the impedance spectroscopy measurements. The software will be available soon, and, of course, to be easily coupled with OSS-DBS.

5) While the authors mentioned that there are some limitations on performance, it is not clear what is the minimal requirement to use the platform for the demonstrated simulations (especially for the docker image). It would be helpful to the reader If the authors can provide the computing time to reproduce the same result with a sample dataset with validated hardware specs (which the hardware environment that authors operate the simulation), as supplementary.

Docker requires a 64-bit processor with at least 4 GB system RAM. No significant difference was observed when running in a Docker container or directly in the system, or in the case when using multiprocessing. Hardware requirements for the platform mostly depend on the desired simulation accuracy that defines the complexity of the FEM problem. Large memory consumption was an issue for adaptive mesh refinement, especially when using direct solvers. The problem was circumvented by application of FEniCS MPI, which, however, should be employed with caution as it might lead to poor solver convergence if the stiffness matrix is highly inhomogeneous. Furthermore, we can't guarantee MPIs proper functioning on every architecture.

Therefore, MPI was disabled when solving the benchmark models. The simulations we conducted in the Docker container on an Intel Xeon(R) Gold 6136 CPU @ 3.00 GHz x 48 machine with 376.6 GB of RAM, with only 1 core and up to 128 GBs used by the platform. The adaptive mesh refinements for the human and the rat brain tissue took 1 hour 51 minutes and 4 hours 15 minutes, respectively. The computational time for the models described in section *Evaluation of Physics in Volume Conductor Model* depends on the amount of available CPUs, as they use parallelization when solving the FEM system in the frequency spectrum and for IFFT. Examples with lower hardware requirements are presented in the tutorial.

A comment regarding MPI for adaptive mesh refinement was added to the manuscript. Hardware specs and the simulation time info was added to S2 Appendix.

6) Regarding the parallel computing in the frequency domain, Have the authors evaluate how much the computing time improved (by %) after parallelization compared to the serial processing? If so, do authors have any recommendations on this setting?

Strong scalability could be observed for the computations in the frequency spectrum and IFT. This is due to the fact that the conducted computations are independent (reciprocal influences are neglected), and thus there is no inter-communication during the run, and the outputs are saved in different files or different datasets if .h5 files are used. For the NEURON simulations, a total average scalability was observed, as some post-processing routines are always conducted with one process. In general, we recommend to use all available and free physical CPUs for the best performance for problems with low memory requirements, otherwise the choice is limited by the available RAM.

For the MPI during adaptive mesh refinement, the scalability is defined by the conditioning of the stiffness matrix and the applied solver. In our experience, the optimal number of CPUs should be chosen empirically.
A comment regarding the recommendation of CPU number for parallelization was added to the manuscript, and a performance comparison was added to S1 Appendix.

Other corrections:

- The title was changed to emphasize the scope of the study and to avoid correlation with a recently published article “FEMfuns: A Volume Conduction Modeling that Includes Resistive, Capacitive or Dispersive Tissue and Electrodes” by Vermaas et al in *Neuroinformatics*.
- Section *Parallel Computations in Frequency Spectrum* was changed to *Parallel Computations in OSS-DBS* as parallelization is also used for inverse Fourier transformation and NEURON simulations.
- The project’s links were updated. Now they direct to the publicly accessible repository on GitHub.
- The numbers in the sentence describing the results for the voltage-controlled simulation were rounded to make it more readable.
- The aforementioned article “FEMfuns: A Volume Conduction Modeling that Includes Resistive, Capacitive or Dispersive Tissue and Electrodes” was addressed in section *Discussion*.
- Appendices were added to meet the reviewers requests.

M.Sc. Konstantin Butenko
Doctoral Researcher in CRC 1270 ELAINE
Institute of General Electrical Engineering
University of Rostock
